# “It Makes You Feel Alive and Younger…but It’s Stressful …My Back and Legs Ache”: A Focus Group Study Encouraging Resistance Training Around Retirement

**DOI:** 10.1177/07334648231193562

**Published:** 2023-09-12

**Authors:** Rachael Frost, Anna Lowe, Snehal M. Pinto Pereira

**Affiliations:** 1 Department of Primary Care and Population Health, 4919 University College London, London, UK; 2 The Advanced Wellbeing Research Centre, 7314 Sheffield Hallam University, Sheffield, UK; 3 Division of Surgery & Interventional Science, Institute of Sport, Exercise and Health, 4919 University College London, London, UK

**Keywords:** exercise, resistance training, retirement, qualitative, sarcopenia

## Abstract

Muscle weakness is a key component of age-related conditions such as sarcopenia and frailty. Resistance training is highly effective at preventing and treating muscle weakness; however, few adults meet recommended levels. Retirement may be a key life-stage to promote resistance training. We carried out a virtual focus group study to explore motivators and barriers to resistance training around the time of retirement, with the aim of determining strategies and messages to increase its uptake. The five focus groups (*n* = 30) were recorded, transcribed and thematically analysed. We found that resistance training was positively viewed when associated with immediate and long-term health and wellbeing benefits and had a social dimension; but there was a lack of understanding as to what constitutes resistance training, the required intensity level for effects; the role of pain; and the consequences of muscle weakness.

**What this paper adds**

• The negative consequences of muscle weakness and benefits of resistance training are not well understood at the point of retirement, even by those who are physically active.
• There is an urgent need to increase awareness and visibility of resistance training at the point of retirement.
• Clear messaging is needed on the type and intensity of exercise required for effects.

**Applications of study findings**

• Public health exercise campaigns should focus on communicating the importance of resistance training and the type and intensity required for benefit at older ages.
• Existing social norms regarding resistance training at the point of retirement need to be challenged.
• Suggested ways to promote resistance training included social media, videos and personal stories.

## Introduction

Muscle weakness increases in prevalence with age (Skelton & Mavroeidi, 2018) and is a key component of age-related conditions such as sarcopenia and frailty (Cruz-Jentoft & Sayer, 2019; Keevil & Romero-Ortuno, 2015). As the global population ages (World Health Organisation, 2015), it becomes critical to prevent/delay muscle weakness to ensure that people maintain the physical capability to independently conduct activities of daily living for as long as possible.

Resistance training encompasses activities to increase/maintain muscle strength. It includes exercising with free weights, resistance machines or resistance bands and bodyweight exercises (Department of Health and Social Care, 2019). Robust evidence demonstrates that resistance training improves strength in middle-aged and older adults (Hurst et al., 2022; Talar et al., 2021), with additional benefits to social functioning and mental health (Khodadad Kashi et al., 2022). Other interventions (e.g. pharmacological, dietary) to improve strength currently have inconsistent findings (Veronese et al., 2019; Yoshimura et al., 2017). UK public health guidelines therefore recommend undertaking muscle strengthening activities at least twice a week (Department of Health & Social Care, 2019), such as resistance training, aerobics and circuit training, racquet sports and to a lesser extent yoga and Tai Chi. In the UK, resistance training activities range from those offered in private or council sports centres and gyms to exercise apps on mobile phones, with free outdoor equipment sometimes available in parks. The range of equipment (e.g. free weights and machines) and classes (e.g. ‘Lift’ and Tai chi) available varies by area and type of facility.

However, along with balance training, muscle strengthening actives are often referred to as ‘forgotten guidelines’ (Strain et al., 2016). On average only 17.3% of adults across 28 European countries meet recommended levels of muscle strengthening activities by doing exercises such as squats (Bennie et al., 2020). When including a wider range of activities (e.g. swimming, aerobics), only 24–31% of Scottish adults met recommended guidelines (Strain et al., 2016). UK policy consequently places greater emphasis on strength training (Department of Health & Social Care, 2019), but studies still demonstrate inadequate understanding of these guidelines (BritainThinks, 2020; Gluchowski et al., 2022).

It is challenging to change physical activity habits. However, key life transition points such as retirement provide an opportunity to increase participation in strength activity, as physical activity levels can increase or decrease substantially, depending on previous occupational activity levels and various lifestyle factors (Skelton & Mavroeidi, 2018; Socci et al., 2021). Retirement offers increased time and energy for activity, while exercise can provide structure and routine (McDonald et al., 2015; Spiteri et al., 2022). Moreover, exercise interventions at this life-stage may confer greater benefits to subsequent physical functioning and independence (Brown & Covinsky, 2020).

Retirement therefore presents an opportunity to prevent/delay loss of independence through building in resistance training at a time of changing routine. Previous studies have identified facilitators to activity at the time of retirement, such as health and social benefits; and barriers including limited time and lack of value placed on recreational exercise (Barnett et al., 2012). However, these studies focused on physical activity, with little mention of resistance training. Given the potentially enormous benefits of resistance training at this life-stage, we aimed to understand motivators and barriers to resistance training around the time of retirement, in order to determine strategies and messages to increase its uptake.

## Methods

We used a focus group approach to elicit individual experiences and social norms around resistance training (Kitzinger, 1995), from a constructivist perspective (Lincoln et al., 2018). We recruited through circulating posters using social media (e.g. Twitter, Instagram), community organisations (e.g. local Age UKs), local older people’s networks and two large workplaces (a large insurance company and university). Potentially interested respondents were emailed study information, a consent form and a demographics questionnaire. Recruitment documents were reviewed by Patient and Public Involvement (PPI) contributors.

Inclusion criteria were UK residents who self-identified as planning to retire in the next two years, retired in the previous two years or were in the process of retiring (‘retiring now’) to ensure we captured experiences and attitudes before and after retirement whilst they are still relevant and memorable. We did not have any age-related restrictions, but excluded those who were semi-retired (different nature of transition) or who had been advised against resistance training by healthcare professionals. We aimed to purposively sample based on gender, ethnicity, current/previous job type, those who did/did not resistance train (yes/no and type) and those who did/did not do other exercise (yes/no and type) through collecting self-reported questionnaire data beforehand. We aimed for focus groups to be mixed in order to promote discussion. In later stages of our study, we targeted recruitment specifically at women and those who did not do resistance exercise as these groups were under-represented.

Virtual focus groups were held March-May 2022 and followed a topic guide developed based on the literature and PPI input, with further probes added as data collection progressed. We did not provide participants with a definition of resistance training beforehand or at the start of the groups, as we wanted to explore their understanding of this. Only after this exploration, we provided the UK Department of Health and Social Care definition. Topics discussed included defining resistance training, barriers and facilitators and changes at retirement (see Appendix 1). For two groups we included a card sort to encourage discussion about the impact of different exercises on strength.

Focus groups were facilitated by RF, an experienced qualitative researcher in ageing, and co-facilitated by SMPP, a physical activity epidemiologist. In two focus groups participants also used the chat function (discouraged in subsequent groups as it slowed real-time discussion and created separate conversations). We video-recorded focus groups using MS Teams and audio was transcribed. Chat comments were inserted into the transcription by researchers at the approximate timepoint of posting. We also made brief field notes after each focus group. We carried out reflexive thematic analysis (Braun & Clarke, 2022). RF and SMPP read all transcripts. RF coded all transcripts using NVivo 12 and developed an initial thematic outline, which was discussed with SMPP. After discussion, RF refined the themes and wrote these up with input from SMPP and AL (a physiotherapist with qualitative and physical activity expertise). PPI contributors reviewed a summary and highlighted areas to focus on and key implications.

We received ethical approval from the University College London Research Ethics Committee (ref 14097/004). All participants provided digital written informed consent (scans of signed forms or digitally signed forms) to participate and received a £20 Marks and Spencer or Amazon voucher for participating.

## Results

Sixty-nine people expressed interest in participating; 15 did not respond to the study information package, eight were ineligible (outside the retirement window or semi-retired), and two declined participation. Of the remaining 44, nine consented but were not invited (in order to focus later sampling on women and those who did not resistance train – see methods), two returned a consent form but were unavailable/did not respond and three were invited but did not attend. Consequently, we spoke to 30 people in five focus groups (with 4–8 people each, Tables 1 and 2). The majority of these people were recruited through social media. Focus groups lasted 60–100 min.

**Table 1.** Demographics.

Demographics	*N* = 30
Age (years)	Mean age 56 (range 45–72)
Gender	10 female, 20 male
Ethnicity	12 White
	8 Black/African/Caribbean/Black British
	4 Asian/Asian British
	4 mixed/multiple ethnic groups
	2 other ethnic group
Most recent/current employment	6 education/research
	5 healthcare
	3 finance/insurance
	3 drivers/logistics
	3 engineering
	2 auto technicians
	2 chef
	2 police/security
	1 IT consultant
	1 receptionist
	1 store manager
	1 missing
Retirement status	18 retiring now
	7 planning to retire in the next two years
	5 retired in the last two years
Regions	13 London
	4 North West
	3 Scotland
	3 South East
	2 South West
	2 West Midlands
	1 East Midlands
	1 North East
	1 Wales

**Table 2.** Composition of Focus Groups.

Focus Group	*N*	Resistance Exercise Participation	Other Exercise Participation
#1	4	4 yes (gym weights; weights; pushups and pullups; 40 pushups daily)	2 yes (jogging; gym), 2 no
#2	4	3 yes (resistance machines at the gym; body weight strain and “plastic strains”; elastic strains and pushups), 1 no	3 yes (walking when possible; road walking >10 km/week; swim and walk most days), 1 no
#3	7	6 yes (programme of free weights and weight machines; balance exercises for 40 min some days; weekly use of gym weight machines; pilates twice a week and gym weights; pushups; bench press, pullups and pushups), 1 no	5 yes (swim about 10 km/week plus cycling to work, dog walks and occasional yoga/circuits; situps and jogging; cycling for transport; triathlon (swim, run, bike), walking, skiing, tennis, badminton; biking), 2 no
#4	7	7 yes (weight training and HIIT session; body weight exercise, yoga and pilates; 2 × situps; 2 × pushups; pullups)	3 yes (cycling, swimming, rowing; walking, cycling and swimming; gym), 1 no, 3 missing
#5	8	1 yes (volunteer gardening work including digging and heavy lifting), 7 no	5 yes (cycling, football 5 times/week; running on the spot for 3 min 3 times/day; long walks and periodic jogging; skipping, jogging and cycling; hiking), 3 no

We identified seven themes related to resistance training around retirement: confusion around resistance training; feeling good; resistance training is too demanding; impact upon health; social dimensions of resistance training; the impact of retirement; and promoting resistance training. The collective range of barriers and motivators from these themes are listed in Table 3.

**Table 3.** Motivators and Barriers to Resistance Training Around Retirement.

Motivators	Barriers
Activities that can be built into daily routines	Confusion around what resistance training encompasses
Feeling good after resistance training (e.g. greater energy, looking good)	Confusion around how intense it needs to be
Feeling stronger and/or seeing progression through equipment usage	Confusion about specific benefits of resistance training versus aerobic training
Perceived long-term health benefits (e.g. osteoporosis prevention)	No perceived effects on strength
Positive previous experience of recovering from injury	Feeing too weak to resistance train: Lack of connecting resistance training to improved physical capability
Maintaining functional ability when older	Lacking confidence
A sense of responsibility for one’s own health	Repeated injury from resistance training
Having a social element (e.g. gym, classes)	Fear of pain and injury
Seeing others resistance train	Too stressful
Sharing information with others	Intimidating terminology
Having family support	Lack of awareness of muscle weakness and its implications, for example, sarcopenia
Seeing others thrive in later life through being active (e.g. parents)	Retirement perceived to be a time to relax and not be active
Having resources on the benefits of resistance training and ideas about how to train	Resistance training is not very visible or socially normal
Retirement seen as an opportunity to be more active	

### Confusion Around Resistance Training

Most participants agreed resistance training was about exercising specific muscles to make them stronger.

anything where you’re specifically working with your muscles, either your own body weight or you can use gym equipment as well or add in some weights to squats (P14, F, 61, white, retired)

Specific single exercises (e.g. squats, pushups) were commonly carried out by participants, particularly men. Some went to the gym and used weights or machines, and a few had a trainer. Participants felt that for optimal health effects, resistance training had to involve different body parts and be complemented by other activities (e.g. jogging, stretching).

However, there was substantial confusion and disagreement over what counted as resistance training (even for active participants), and how intensive it should be. Those who were keen on resistance training felt everyday activities were insufficient to build muscle strength as they lacked purpose and intensity.

Everything else we do during the day wouldn’t be considered as exercise in the real sense of it […]Resistance training are done in sets, depending on what you seek to achieve [chat data, P6, M, 45, Black, retiring now]

There was particular confusion over aerobic activities, such as swimming or running, which participants felt included a degree of strength building, suggesting lack of distinction between muscle strength and muscle endurance. Women particularly felt that every bit of strength activity helped, and it was important to build this into daily routines.

things you do in your daily activities could be considered a form of resistance training. Like making sure you carry your shopping home rather than getting the bus or you know, go up and down the stairs. (P14, F, 61, white, retired)

Using everyday activities was seen as the best way to engage less active people, but resistance training was more difficult to build into routines as a useful activity compared to aerobic activities, for example, active travel. With regards to terminology, the terms ‘resistance training’ and ‘weights’ were thought to be potentially intimidating.

put all the normal activities before the using weights etcetera activities might draw in people who as soon as you see weights you think oh blimey, no thanks (P1, F, 59, white, retiring now)

However, few alternatives were suggested that clearly communicated resistance training – participants recommended non-specific terms such as ‘staying physically active’ or ‘exercises’.

### Feeling Good

Participants were most motivated to resistance train when they associated it with feeling good, either through enjoying the activity itself or the immediate post-training benefits, including greater energy, greater stamina and looking good. Feeling good was prioritised more as a reason to exercise for men; women participants tended to focus more on health benefits (see Theme 4).

I do it because I love it[…]I want to wear my shirt and feel good when I’m walking around (P23, M, 60, White, retired)

Feeling stronger was a vital outcome for both genders, although there were mixed views on how quickly this could happen. Those who did not notice results ceased exercising fairly quickly, whereas those who noticed a clear difference in their strength were much more motivated to continue.

My legs, I had more muscles on my leg and I felt myself, I felt stronger…that is one of the things that has been motivating me to keep on doing the squats. (P39, M, 52, Black, planning to retire)

Using equipment could facilitate faster and better results through providing greater challenge; it also more obviously demonstrated progression. Participants who favoured equipment often preferred gym-based training or had a specific purpose for training.

If you want to just look good and fit, equipment wouldn’t be of utmost importance […]If you want to be ripped/shredded then equipment would be your things [chat data, P6, M, 45, Black, retiring now]

When participants’ aims were to feel subjectively stronger and more energetic rather than ‘ripped’, training with their own body weight was easier, cheaper, more convenient and seemed to achieve the desired results. Resistance bands were only mentioned in the context of physiotherapy for an injury.

There was general agreement that resistance exercise improved mental wellbeing during or shortly after training, even for those who found it stressful.

It makes you feel alive and younger a bit, but it’s stressful though, and my back and legs ache from it. (P22, M, 52, White, retiring now)

Finally, yoga was viewed positively as a gentle way to gain/maintain strength whilst relaxing, and was undertaken by mainly female participants. However, the ability to continuously challenge muscles with yoga was not considered.

For me, it’s still yoga and my walking because as a retired somebody you’ve just gone through a lot of stress during the early days and you just want to rest and have a relieve of things (P45, F, 50, white, retiring now).

### Resistance Training is Too Demanding

Others were unmotivated to do any resistance training or had noticed this in their contemporaries, due to a lack of confidence, lack of enjoyment and feeling unable to do this kind of exercise. Work or other leisure activities were prioritised instead. These participants struggled to identify ways in which people could be motivated to resistance train; they did not connect resistance exercise with improved physical capability.

I don’t think there can be much of anything to make people exercise […] Someone like myself I definitely wouldn’t want to because I don’t think I have the stamina and leg strength for that. (P40, M, 55, Asian, retiring now)

One factor clearly affecting engagement with resistance training was pain. Pain was typically discussed in the context of the response of muscles to loading, either during or shortly after training. Pain was viewed positively and as necessary for results by those who resistance trained:

when you shred your body, it’s shredding weakness. (P3, M, 60, Black, retiring now)

For those less keen on resistance training it was undesirable and stressful.

I don’t exercise that much because it’s kind of very stressful and it makes me weak, my muscles ache (P45, F, 50, white, retiring now)

Aerobic activities (including walking, jogging, cycling) were viewed as less stressful than resistance training.

### Impact Upon Health

Health was a crucial factor in participants’ decisions as to whether to resistance train, particularly (but not exclusively) for women. Long-term health benefits were strongly motivational, such as helping with musculoskeletal issues or preventing the onset of obesity and diabetes. However, resistance training benefits were often conflated with aerobic training benefits (e.g. for cardiovascular health). Preventing osteoporosis was particularly salient for female participants.

with women going through the menopause or after the menopause, we have to be so careful with our bone density, so like P5 was saying, we do need to do some form of resistance training to try and keep ourselves reasonably healthy and not so prone to breaks and things should we fall. (P16, F, 65, white, retiring now)

As most participants were at mid-life to early late life, health issues (e.g. hospitalisation due to Covid) stopped participants exercising only temporarily. A few had previously used resistance training to recover from injury or musculoskeletal pain, leading to positive views.

it took a year of intense resistance training really [after back surgery], with physio advice, to get to a point where it is now stronger than it was before. So, it really was of benefit, big style. (P31, M, 66, white, retired)

Combating the effects of ageing was important to some participants, who feared losing their functional abilities. More active participants feared losing an active lifestyle, but most participants were concerned with maintaining day-to-day mobility and functioning for as long as possible.

I just want to be able to move for as long as possible and ideally not fall down and break something. (P1, F, 59, white, retiring now)

This was underpinned by a sense of responsibility for one’s own health, and not wanting to depend on the National Health Service (NHS), particularly from those who were more active.

I just want to encourage the gentleman who doesn’t feel motivated, please, think about this as your responsibility of taking care of yourself and getting stronger and happier (P2, F, 68, Mixed, retired)

There was limited reference to preventing muscle loss, sarcopenia or feelings of weakness either at present or further ahead in retirement. Although some older participants had recognised manifestations of weakness (e.g. difficulty with stairs) and connected this to resistance training, for younger participants feeling weak was instead considered a reason not to do resistance exercise.

Resistance training was also considered more hazardous if done incorrectly than other exercise, and some had previously experienced injury. Where they had successfully adjusted their exercise (e.g. reduce weight, switch to lower intensity exercise), they continued to train; however, multiple injuries or pain after different exercises deterred a few participants from doing future resistance exercise.

I had an extremely bad experience of pilates, physio and both, yes with the NHS, in which I have actually been damaged. So I am now very, very wary of exercises. (P11, F, 65, white, retiring now)

### Social Dimensions of Resistance Training

Many participants enjoyed a social element to their exercise, more commonly for outdoor aerobic activity (e.g. walking, jogging). Strength activities were not prioritised compared to these types of activity, although for a few participants having sufficient strength to lift grandchildren acted as a motivator. To our participants social resistance training meant attending classes or being in the gym, with classes particularly favoured by women. Exercising with others reduced boredom, provided motivation, committed a person to attend and was a means of social interaction.

I’ve watched some online videos and I’ve used most apps that’s for the yoga too but it became too boring when I was all alone doing it. (P44, F, 57, Mixed, planning to retire)

Classes were not preferred by all participants however; they required more effort to attend and were less convenient and affordable than home-based exercise. They could also generate competitiveness, which was motivational for more confident and active participants but intimidating for less active participants and came with a risk of pushing themselves to the point of injury. Participants recommended greater targeting by ability in resistance classes, to avoid excessive challenge/slowness, and did not like the idea of targeting by age due to the inherent heterogeneity in fitness within similar age groups. For a small number of people, the gym offered similar motivational and social benefit through companionship and competition, with more flexibility than classes.

Yes, exactly, P19 […] seeing others do the same thing I’m doing or even more, it keeps me going, so actually I prefer the gym to my house. (P23, M, 60, white, retired)

However, gyms could be perceived as intimidating or male-oriented by those who did not use them, although this seemed to rarely be borne out in practice. Gyms had additional barriers such as being indoors, too costly or the exercise there lacking a purpose. For those who did not like the gym or classes, there was a particular lack of visibility elsewhere of others doing resistance training. It was thought to be less acceptable to do publicly than activities such as jogging.

you don’t visibly see people [resistance] exercising much, I mean I know of it, I have seen it advertised but I have not actually seen it happening. (P11, F, 65, white, retiring now)

For those preferring home exercise, not seeing others exercise could be overcome through sharing information or videos about exercises and what works for them.

when we meet up […]somebody will say, you know, “Well, my physio said this is what I’ve got to do.” And actually it’s just an exchange of ideas and you think, Well, actually, when nobody’s watching I’m going to try and see if I can even lift a tin of baked beans [laughs] (P13, F, 72, Asian, retired)

Some reported training with family members, such as partners, adult children or grandchildren, at home. This provided motivation and was a source of instruction and knowledge. Exercising with family was viewed as less intimidating and competitive than with strangers or friends.

my son-in-law, [name], we both do a little jogging, and from the jogging we hit the gym together. (P23, M, 60, white, retired)

A few participants had tried training with apps, but they lacked the desired social dimensions and individualised feedback on whether an exercise was being performed correctly.

### The Impact of Retirement

Retirement was seen as an opportunity to become more active through greater time and freedom for enjoyable activities, but not for specific health-related exercises or resistance training.

it’s just so soon after retirement I am still just going out so much socially. I am just cramming loads of sport into the day, seeing my mates, going out to gigs, just travelling and having fun. (P1, F, 59, white, retiring now)

Those less keen on exercise still viewed retirement as an opportunity for activity, with positive effects on stamina. Those who exercised only when they felt like it, had not, or did not plan to change their routine. Some participants felt resistance training was too stressful for retirement, whilst others had noticed in their peers a desire for sedentary retirement and relaxation rather than exercise. A few reported motivation to start or continue exercising by seeing inactive peers experiencing more health problems, or through seeing others live an active life at older ages (e.g. parents).

A small number of people suggested pre-retirement information may encourage uptake of resistance training. Although retirement was an opportunity for greater activity, it did not seem to be as salient for other health-related lifestyle changes (e.g. diet, sleep), which tended to be prompted by negative health events, such as injuries and illness.

I tripped up getting off a train and broke my ankle, so the doctor sent me for a bone density scan. I was diagnosed with osteopenia, which was a huge wake up call, because I considered myself fairly fit. (P5, F, 53, White, retiring now)

### Promoting Resistance Training

The main channel mentioned for promoting information about resistance training was social media, particularly for those not already interested. A few others mentioned billboards, posters, leaflets, webinars and courses. Promotional materials needed to show how others like them, or recognised authorities/celebrities, had trained and seen results.

Maybe a social media page where people who have undergone resistance training can come and give their testimonies on how the resistance training actually helps them (P19, M, 50, Black, planning to retire)

Only a small number felt the NHS should promote resistance training, chiefly because people around retirement were starting to use health services more frequently. There was little reference to NHS professionals promoting resistance exercise (other than physiotherapists), and more of a sense that this was a self-care activity (see Theme 4).

A few participants emphasised the importance of knowing how to train safely, particularly with musculoskeletal conditions. Personal trainers were considered valuable sources of information by the few participants who used them.

Once people were interested in resistance training, YouTube videos were valued as they showed participants how to do an exercise and allowed sharing with others. Google or search engines were more often used to find classes or gyms, or types of exercise.

I started when my children came around […]they told me I should be doing some push-ups, and then I went on the internet and I saw some people doing push-ups. (P24, M, 50, White, retiring now)

## Discussion

In our five focus groups with 30 participants around retirement, we found that resistance training was positively viewed when associated with immediate and long-term health and wellbeing benefits and had a social dimension. Key barriers were lack of clarity on what resistance training includes, lack of awareness of muscle weakness and its consequences and the role of pain and potential for injury. Ways to promote resistance training included social media, videos and personal stories, to achieve widespread awareness and visibility and improve social norms around resistance training. Table 4 summarises our key implications.

**Table 4.** Implications From This Work.

Key Finding	Implications
Lack of clarity on (i) what resistance training is and (ii) how intense resistance training needs to be/feel like	Provide education on ways to start building strength, for example, by doing activities of daily living (e.g. stair climbing) and yoga/tai chi. Once stronger, to progress in strength, it is vital to encourage a focus on setting time aside specifically for resistance training with bodyweight, resistance bands or weights. Improve understanding that putting muscles under stress is necessary to build strength and see results: Provide clear guidance on what this feels like as people rely on subjective feedback from their own body. There is a need for personalisation (dependant on current activity/fitness levels) and information on and provision of a range of resistance training options (e.g. with bodyweight, resistance bands or weights). This should be co-designed with specific groups in order to provide more tailored messages. Resistance training needs to be more visible and accessible at the point of retirement and beyond. Outdoor classes or training activity, social media posts and videos may help to normalise resistance training. Improve healthcare professionals’ communication of resistance training messages to the public (with respect to frequency, intensity, etc).
Lack of awareness of muscle weakness	Around the time of retirement improve (i) awareness of the negative impacts of muscle weakness and its consequences, (ii) information on early signs of muscle weakness (for example, PPI contributors indicated difficulty opening a car door), (iii) the role of resistance training in prevention and treatment of muscle weakness (which includes discussion around the risk of injury) and (iv) the wider benefits of resistance training, for example, mental health, increased independence, etc.
Retirement is a good opportunity to increase activity levels but is not necessarily a time to focus on health-related changes	There is a need to reinforce the importance of strength training activities in health service contacts for all middle to older aged adults. There is a need to provide advice on how resistance training exercises can be built into a sustainable new routine after retirement.

Our study is novel in terms of the target life-stage. Identified barriers and facilitators are similar to those found in studies on resistance training in older populations; mainly, benefits of increasing strength and better mental and physical health; and barriers of poor health, fatigue, lack of willpower and availability of appropriate facilities/exercise options (Burton et al., 2017; Henwood et al., 2011). We extend current literature by highlighting that the younger demographic we examined were more concerned with the role of pain and less concerned with the risk of cardiovascular events raised by older samples (Burton et al., 2017). Cost barriers cited in another study (McDonald et al., 2015), were rarely raised by our sample. We did not collect socioeconomic status data; however, our population was typically in professional jobs and retiring at younger ages, so are likely to be relatively affluent. Therefore, when considering transferability, not all findings may apply more widely. For example, recreational physical activity (as opposed to purposeful activity, for example, housework/gardening) in retirement tends to be less valued by people in lower socioeconomic classes (Barnett et al., 2012), which is echoed by studies on physical activity messaging to underserved groups (Nobles et al., 2020).

Others have also found a lack of awareness of guidelines and confusion around what counts as resistance training, with most people developing their own routine (BritainThinks, 2020; Cavill & Cowburn, 2019; Gluchowski et al., 2022). Interviews with older adults show a lack of understanding regarding muscle weakness, with cognitive and psychological strategies used to cope with weakness rather than exercise (Rush et al., 2013). Resistance training needs to be performed with a relatively high degree of effort, where muscles feel tense, warm or shaky by the end of the first set of an exercise (Hurst et al., 2022). It is more difficult to build into daily life at a sufficient level than aerobic activity (e.g. via active commuting). In very deconditioned groups, increases in low intensity strength activity (e.g. walking, stair climbing) are likely to produce gains; however, beyond a certain point, gains will be limited due to a lack of progression, and our study suggests this would reduce motivation to continue.

There is a need for multi-pronged programmes to promote strength building activities around the time of retirement. For example, this could include support by celebrity endorsers, promotion by NHS front line staff (e.g. GPs, physiotherapists), and trusted social media information on safe and effective habit changes. It is important to use a range of information sources and authorities to capture different groups (Nobles et al., 2020). Additionally, behaviour change relies on physical capability, opportunity and motivation to change as well as knowledge (Michie et al., 2011). Interventions therefore need to provide opportunities to try resistance training for at least six weeks (suggested as sufficient to begin forming habits (Kaushal & Rhodes, 2015)), during which time they should see benefits, providing ongoing motivation (Henwood et al., 2011). For example, community-based exercise classes show positive impacts on strength and physical function (Levy et al., 2020). Such programmes could be implemented through partnerships with the leisure industry, community groups and workplaces, to offer and promote a wide range of training options.

Strengths of our work include rich data from a diverse range of participants according to ethnicity, retirement point and previous/current job. However, we recruited more participants from professional occupations and struggled to recruit those who did not resistance train. Our focus groups therefore had more participants who resistance trained, which may have led to more homogenous groups or limited the range of discussion. Whilst we set out to explore facilitators of resistance training in those who did train as well as barriers in those who did not, our sample may be more enthusiastic about resistance training than the wider population. Thus, further barriers might be identified in future research in groups who do not train. Importantly, even within those who did resistance train, many were confused as to what it compromises. As we recruited by email and social media, we also limited the sample to those who were familiar with these channels adding an element of selection bias. Different views, particularly around promoting resistance training, may be present in those without email access.

In some groups, participants having their camera off or typing in the chat stilted discussion; however in general, participants did discuss and debate points with each other, share resources and ideas and encourage each other. Our sample, with a mean age of 56 years, reflects a younger demographic than expected (UK retirement age is currently 66) and they may thus be more active. However, our findings are similar to those from Australian and UK adults aged over 65 (Gluchowski et al., 2022; Henwood et al., 2011), suggesting stability of opinions over these ages. In terms of researcher influence on data collection, both researchers who collected data are women who regularly exercise and are younger than retirement age. Although this could have caused participants to talk more positively about resistance training or want to come across as more active, negative views were elicited, along with discussions around some participants being inactive and perceived gender differences.

## Conclusion

Our work indicates that benefits of resistance training and negative consequences of muscle weakness are not well understood at the point of retirement, even in those who are active. Given that resistance training is a highly effective but under-utilised public health intervention to maintain independence and quality of life, there is urgent need to increase awareness, particularly regarding the intensity needed and the range of exercise options available. There needs to be greater parity in physical activity guidelines between resistance and aerobic training. Encouraging visibility of resistance training in public, on social/mass media and ensuring ways to train socially are available will help to communicate information and normalise resistance training. Further research to develop and test effective ways to provide information on and improve uptake of resistance training at the point of retirement is vital.

## Supplemental Material

Supplemental Material - “It Makes You Feel Alive and Younger…but It’s Stressful …My Back and Legs Ache”: A Focus Group Study Encouraging Resistance Training Around Retirement Supplemental Material for “It Makes You Feel Alive and Younger…but It’s Stressful …My Back and Legs Ache”: A Focus Group Study Encouraging Resistance Training Around Retirement by Rachael Frost, Anna Lowe, and Snehal M. Pinto Pereira in Journal of Applied Gerontology.
