# Supplementary material for: “It Makes You Feel Alive and Younger…but It’s Stressful …My Back and Legs Ache”: A Focus Group Study Encouraging Resistance Training Around Retirement
Source: J Appl Gerontol. 2023 Sep 12;43(1):59–68. doi: 10.1177/07334648231193562 (PMC10693719; doi:10.1177/07334648231193562)
Supplement: Supplemental Material - “It Makes You Feel Alive and Younger…but It’s Stressful …My Back and Legs Ache”: A Focus Group Study Encouraging Resistance Training Around Retirement [file sj-pdf-1-jag-10.1177_07334648231193562.pdf]

Topic Guide: Staying strong!

Thank you for taking part in this focus group today. The focus group will discuss resistance training. At older ages, muscle weakness is a major problem. It leads to falls, hospitalisation and loss of independence. One of the best ways to prevent this is to do resistance exercises, but there is low awareness and uptake of these at all stages of life. We would therefore like to understand your perceptions and experiences of resistance training, barriers and facilitators to this and how we might encourage people to take this up at the point of retirement.

We would like to hear everyone's views, including positive and negative views and experiences around resistance training. If you talk a lot, please remember to give others in the group a chance to speak. If you do not speak much, we would really appreciate you coming forward and letting us know your views. We also ask that everyone respects the privacy of others in the group and doesn't discuss the content of the focus groups outside the group.

We would like to audio record the discussion so we can accurately capture what has been said, but will ensure that any names and locations are removed or changed when we transcribe the recording. If you decide you no longer want to take part, you can leave at any time without giving a reason. As it is difficult to remove data once part of the focus group – we cannot always tell who was speaking on the recording – you will not be able to remove any data recorded up to that point.

Do you have any questions? If everyone is happy, we will <u>start the recorder</u>.

1. **Card sort**

We will start with an activity. Here are four cards with different exercises shown on them. Have a little think about how you would rate these from the ones most likely to benefit muscle strength to the least. Would any like to explain their thinking?

2. **Resistance training (if not already covered)**

What would you consider as resistance training?

- Prompts: weight lifting, using resistance bands, bodyweight (squats, lunges) anything else?
- What about everyday activities, e.g. picking up grandchildren, lifting heavy shopping

Current guidance states that activities to improve strength should ideally use major muscle groups in the upper and lower body and be repeated until the muscles are tired out and unable to repeat the exercise for a short period of time. This includes activities like using the weight of your own body, free weights, resistance machines or resistance bands. Some everyday activities can also improve strength, such as lifting children or heavy shopping, gardening, stair climbing or wheeling a wheelchair. It's advised that activities are carried out twice a week, but any strengthening activity is better than none.

What do you think about this? Is anything new or surprising?

If asked about: Emerging evidence suggests that such activities performed just once a week at a higher volume of work can also provide similar health effects, but at this stage the evidence is insufficient to justify changing the frequency recommended.

3. Barriers and facilitators

Do you do any of these activities that count as resistance training?

- What?
- Why/why not?
- How often?
- When?
- Where?
- Who with?
- When did you start doing these? What prompted you to start?
- What prompts you to keep doing these?
- Have you ever done these? Why did you stop?

What kind of equipment do you use?

- How, why, when?
- Where did you learn how to use this?

Would you ever consider doing these activities?

- Why/why not?
- What kinds of activities?

What would make you decide to do/do more resistance training?

- Prompts: benefits, as a social activity, knowledge of what to do, others doing it/advising
- If you noticed you had strength difficulties (e.g. opening jars, car door) what would you do?

What might put you off doing resistance training/make you stop?

- Prompts: time, money, need to join a gym, knowledge, potential for damage/pain

Does anyone you know do resistance exercise?

If I asked you to picture someone who did resistance training, what would you think of?

4. Retirement

For those who have retired, has the amount you exercise changed? In what way?

For those planning to retire, are you planning to change the amount you exercise? In what way?

Are there are other changes you thought about making to benefit your health?

- Prompts: diet, socialising, hobbies

How might we encourage people to take up resistance exercise at the point of retirement?

Where would you like to find out more information on resistance training?

- Prompts: internet (what kind of websites?), radio, TV, book, gym

If you're thinking about starting a new form of exercise, how do you go about this?

Thank you for your time today. [Summarise topics covered] Is there anything else we haven't covered that you would like to mention?

Thanks everyone. We will send you a £20 voucher/e-voucher within the next week (preference for amazon or M&S?). We will use the results from these groups to develop an infographic and video promoting resistance training at older ages. We will send you a copy of these once developed, as well as a summary of what we have found from all the groups.

If you know anyone who might be interested in taking part, please pass on information about the study!

COREQ (COnsolidated criteria for REporting Qualitative research) Checklist

A checklist of items that should be included in reports of qualitative research

**Topic**	**Item No.**	**Guide Questions/ Description**	**Author Responses**
**Domain 1: Research team and reflexivity**			
Personal Characteristics			
Interviewer/facilitator	1	Which author/s conducted the interview or focus group?	XX, with YY as co-facilitator (Methods, para 4)
Credentials	2	What were the researcher's credentials? E.g. PhD, MD	All researchers have PhDs.
Occupation	3	What was their occupation at the time of the study?	Methods, para 4<br>XX is a Senior Research Fellow<br>YY and ZZ are Associate Professors
Gender	4	Was the researcher male or female?	All researchers are female (p. 21)
Experience and training	5	What experience or training did the researcher have?	XX has substantial experience leading and working on multiple qualitative research projects, and teaches qualitative methods to post-graduates. Her work focuses on ageing.<br>YY is an epidemiologist with expertise in physical activity.<br>ZZ is a physiotherapist with expertise in qualitative work and physical activity with under-represented groups.<br>A brief summary of this is in the Methods (para 4)
Relationship with participants			
Relationship established	6	Was a relationship established prior to study commencement?	No relationship with individual participants was established prior to study commencement, although researchers had previous relationships with some of the recruiting organisations.
Participant knowledge of the interviewer	7	What did the participants	We briefly introduced ourselves as

Topic Guide: Staying strong!

		know about the researcher? e.g. personal goals, reasons for doing the research	researchers interested in this field.
Interviewer characteristics	8	What characteristics were reported about the inter-viewer/facilitator? e.g. Bias, assumptions, reasons and interests in the research topic	We briefly report this in the discussion (Discussion, final para). All researchers have the perspective that increased exercise is positive, and assumed that retirement would be a good opportunity for increasing this.
**Domain 2: Study design**			
Theoretical framework			
Methodological orientation and Theory	9	What methodological orientation was stated to underpin the study? e.g. grounded theory, discourse analysis, ethnography, phenomenology, content analysis	Methods, para 1.
Sampling	10	How were participants selected? e.g. purposive, convenience, consecutive, snowball	Methods, para 2
Method of approach	11	How were participants approached? e.g. face-to-face, telephone, mail, email	Methods, para 1
Sample size	12	How many participants were in the study?	Results para 1
Non-participation	13	How many people refused to participate or dropped out? Reasons?	Results para 1
Setting			
Setting of data collection	14	Where was the data collected? e.g. home, clinic, workplace	Methods, para 3
Presence of non-participants	15	Was anyone else present besides the participants and researchers?	A small number of participants had background noise or left briefly for an interruption, but otherwise we could not easily determine this due to the remote nature of the group.
Description of sample	16	What are the important characteristics of the sample? e.g. demographic data, date	Tables 1 and 2
Data collection			
Interview guide	17	Were questions, prompts, guides provided by the authors? Was it pilot tested?	Methods para 3, appendix 1
Repeat interviews	18	Were repeat interviews carried out? If yes, how many?	No
Audio/visual recording	19	Did the research use	Yes. Visual recording was used but

		audio or visual recording to collect the data?	only audio was transcribed and analysed, and participants could choose whether to have their camera on (Methods, para 4)
Field notes	20	Were field notes made during and/or after the interview or focus group?	Methods, para 4
Duration	21	What was the duration of the interviews or focus group?	Results, para 1 (60-100min)
Data saturation	22	Was data saturation discussed?	After the first three focus groups we noted recurring themes for exercisers and men and so sampled more widely. By the end of the fifth focus group, we noted further recurring themes.
Transcripts returned	23	Were transcripts returned to participants for comment and/or corrections?	No, we did not do this.
**Domain 3: analysis and findings**			
Data analysis			
Number of data coders	24	How many data coders coded the data?	1 (RF, see Methods para 4)
Description of the coding tree	25	Did authors provide a description of the coding tree?	No (insufficient space)
Derivation of themes	26	Were themes identified in advance or derived from the data?	Derived from the data (see Methods para 4)
Software	27	What software, if applicable, was used to manage the data?	NVivo 12 (see Methods para 4)
Participant checking	28	Did participants provide feedback on the findings?	No. We sent participants a summary of the findings after the study but did not request formal feedback. Two PPI members reviewed the findings.
Reporting			
Quotations presented	29	Were participant quotations presented to illustrate the themes/findings? Was each quotation identified? e.g. participant number	Yes (see all Results)
Data and findings consistent	30	Was there consistency between the data presented and the findings?	Yes (see all Results)
Clarity of major themes	31	Were major themes clearly presented in the findings?	Yes (see all Results)
Clarity of minor themes	32	Is there a description of diverse cases or	Yes (see all Results)

		discussion of minor themes?	

Developed from: Tong A, Sainsbury P, Craig J. Consolidated criteria for reporting qualitative research (COREQ): a 32-item checklist for interviews and focus groups. *International Journal for Quality in Health Care*. 2007. Volume 19, Number 6: pp. 349 – 357

Additional Guidelines for Completing the COREQ Checklist for *Journal of Applied Gerontology*:

- This checklist will be published online as supplementary material and we require it to be in the form of a publishable table. Please make sure that material does not bleed outside of cells, etc.
- This checklist is designed to direct readers to relevant material in the manuscript. Where applicable, please direct readers to various sections of the manuscript, such as a Methods section, Conceptual Framework, table or figure. Pages may shift during the publication process so please avoid directing readers to specific page numbers.
- This checklist also is designed to supplement information that may not be reported in the text and/or provide additional details related to information that is reported in the text.

Once you have completed this checklist, please save a copy and upload an anonymized version of it as part of your *Journal of Applied Gerontology* submission. DO NOT include this checklist as part of the main manuscript document. It must be uploaded as a separate supplemental file. If the paper is accepted, a non-anonymized version should be provided with the final submission of the main manuscript.
